# Next-generation sequencing of experimental mouse strains

**DOI:** 10.1007/s00335-012-9402-6

**Published:** 2012-07-07

**Authors:** Binnaz Yalcin, David J. Adams, Jonathan Flint, Thomas M. Keane

**Affiliations:** 1 Center for Integrative Genomics, University of Lausanne, Lausanne, Switzerland; 2 Institute of Genetics and Molecular and Cellular Biology, 67404 Illkirch, France; 3 Wellcome Trust Sanger Institute, Hinxton, Cambridge, CB10 1HH UK; 4 Wellcome Trust Centre for Human Genetics, Roosevelt Drive, Oxford, OX3 7BN UK

## Abstract

Since the turn of the century the complete genome sequence of just one mouse strain, C57BL/6J, has been available. Knowing the sequence of this strain has enabled large-scale forward genetic screens to be performed, the creation of an almost complete set of embryonic stem (ES) cell lines with targeted alleles for protein-coding genes, and the generation of a rich catalog of mouse genomic variation. However, many experiments that use other common laboratory mouse strains have been hindered by a lack of whole-genome sequence data for these strains. The last 5 years has witnessed a revolution in DNA sequencing technologies. Recently, these technologies have been used to expand the repertoire of fully sequenced mouse genomes. In this article we review the main findings of these studies and discuss how the sequence of mouse genomes is helping pave the way from sequence to phenotype. Finally, we discuss the prospects for using de novo assembly techniques to obtain high-quality assembled genome sequences of these laboratory mouse strains, and what advances in sequencing technologies may be required to achieve this goal.

## Introduction

In recent years, DNA sequencing has undergone a revolution through the development of much higher throughput sequencing technologies resulting in a significant reduction in the cost per base pair (Turner et al. 2009). We have reached the point where it is now possible to sequence the entire genome of a mammalian species for just a tiny fraction of what it cost to generate the raw sequencing data for the mouse reference genome. These second-generation sequencing technologies such as Illumina (Bentley et al. 2008), Roche/454 (Margulies et al. 2005), and SOLiD (Shendure et al. 2005) are based largely on the same principle: sequencing many millions of DNA fragments in parallel (Turner et al. 2009). The sequencing reads produced by these technologies are generally much shorter than capillary sequence reads, a factor that conflates the challenge of analyzing large mammalian genomes (Pop and Salzberg 2008).

We used second-generation sequencing technologies to deeply sequence 17 mouse strains on the Illumina platform (Keane et al. 2011; Yalcin et al. 2011). In this review we describe the different types of sequence variation uncovered, with specific emphasis on structural variation, and discuss the implications of our findings for understanding how sequence variation influences phenotypic differences. Finally, we examine the prospects for using second- or third-generation sequencing technologies to create improved high-quality (Chain et al. 2009) genome sequences for these mouse strains.

## Identification of SNPs and short indels

The raw sequence for our study of the 17 mouse strains was generated on the Illumina GAII platform (Bentley et al. 2008), with reads of between 54 and 108 bp generated from both ends of DNA fragments of 300–500 bp in size. When these reads were aligned to the reference strain (C57BL/6J; MGSCv37 assembly), 13–23 % of the reference genome assembly could not be confidently accessed due to the presence of highly divergent sequence or high copy-repeated sequences that were longer than the sequence reads and fragment size (such as transposable elements, telomeric repeats, centromeres, or low-complexity regions) (Flicek and Birney 2009).

In the mouse genome, and indeed in other vertebrate genomes, the simplest and most prevalent type of molecular variation is the single nucleotide polymorphism (SNP). The algorithms for calling SNPs scan across the reference genome observing the aligned read bases at each position, and then use read depth and base quality to identify sequence mismatches with high accuracy (Pop and Salzberg 2008). Our analysis found a total of 56.7 M SNP sites, but the number of SNPs varied considerably among strains, ranging from just a few thousand in the C57BL/6NJ strain to 35.4 M in SPRET/EiJ. The major denominator for the number of SNPs discovered was the genetic distance of the mouse strain from the reference C57BL/6J genome. A combination of three SNP calling algorithms were used (SAMtools (Li et al. 2009), GATK (McKenna et al. 2010), and QCALL (Le and Durbin 2011)), with the final set of SNPs consisting of sites that were identified by at least two of the callers. In agreement with findings from the human 1000 Genomes pilot project where a majority voting scheme was employed to merge SNP genotypes (1000 Genomes Project Consortium 2010), this strategy was found to minimize the false discovery rate while maintaining high sensitivity. Small insertions and deletions (indels) of 1–100 bp were also detected using a combination of Dindel (Albers et al. 2011) and also by carrying out de novo assembly of the reads and comparing the resulting contigs to the reference genome assembly (Keane et al. 2011). Overall there were approximately six times fewer indels than SNPs, and it was found that the indel calls were of lower sensitivity and specificity than SNP calls owing to the complexity of calling these variants from short read sequences.

The accuracy of SNP and indel calls was established by comparing variant calls to 16.3 Mbp of finished BAC sequences from the NOD/ShiLtJ strain. The NOD/ShiLtJ BAC sequence represented a unique resource of high-quality finished sequence that allowed us to robustly assess our false-negative and false-positive rates. In inaccessible regions, the 13–23 % of the reference genome where we were unable to unequivocally place sequence reads, we found a threefold enrichment for sequence variants, implying that current sequencing technologies miss at least 30 % of sequence variation. However, it remains unclear how much of this missing variation is functional as the inaccessible regions of the genome are replete with low complexity, simple repeats, and high copy repetitive elements such as long interspersed nuclear elements (LINEs). The number of SNP variants we discovered from sequencing the 17 mouse genomes represented a sevenfold increase in the number of these variants in public databases such as dbSNP.

Interestingly, a significant subset of the SNP (0.12 M) and indel (0.005 M) positions discovered in our analysis resulted in amino acid substitutions, highlighting the diversity in coding sequence between mouse strains.

## Identification of structural variation

We defined structural variation as sequence variants that are greater than 100 bp. Structural variants (SVs) are an important source of sequence variation, in both human (Conrad et al. 2010; Feuk et al. 2006; Kidd et al. 2008; Mills et al. 2011; Redon et al. 2006), and mouse (Yalcin et al. 2011; Agam et al. 2010; Akagi et al. 2008; Cahan et al. 2009; Cutler et al. 2007; Graubert et al. 2007; Henrichsen et al. 2009; Quinlan et al. 2010) genomes. They include insertions, retrotransposon elements, inversions, segmental duplications, and other genomic rearrangements.

The extent of structural variation in the mouse genome was first demonstrated using differential hybridization of genomic DNA to oligonucleotide arrays [array comparative genome hybridization (CGH)] (Cahan et al. 2009; Cutler et al. 2007; Graubert et al. 2007; Henrichsen et al. 2009). While array CGH methods can interrogate hundreds of genomes, they are blind to some SV types such as those that are copy-number neutral and rarely provide breakpoint resolution. Furthermore, array CGH is generally unable to detect SVs smaller than 5 kbp and are poorly reproducible between studies (Agam et al. 2010). Previous array CGH analyses estimated that the proportion of the mouse genome affected by SVs ranged from 3 % (Cahan et al. 2009) to over 10 % (Henrichsen et al. 2009).

The greater sensitivity and specificity of next-generation sequencing technologies represent a significant advance on array-based methods of SV identification. Our recent catalog of structural variation contains far more SVs than previously identified (Nellaker et al. 2012; Yalcin et al. 2011). We found structural variants at 281,243 unique sites, amounting to 711,920 SVs in 13 classical and 4 wild-derived inbred strains of mice, affecting 1.2 and 3.7 % of the genome, respectively. The majority of SVs are less than 1 kbp in size, below the level amenable to detection by array CGH methods.

Methods to localize SVs using next-generation sequencing are based on paired-end mapping (PEM) (Medvedev et al. 2009) (also reviewed in (Alkan et al. 2011)): two short paired-reads from both extremities of a segment of DNA (the insert) and at an approximately known distance are sequenced and mapped back to the reference genome. Typically, variation in the expected number of reads mapping to the reference sequence is used to identify copy number variation, while deviations from the expected distance between reads, and the orientation of reads, are used to determine the type of structural variant such as deletions and inversions.

In the past few years, a plethora of software tools have been developed to detect SVs from next-generation sequencing data. These tools exploit (1) read-pair, (2) split-read, (3) read depth, and (4) sequence assembly information. A summary of software tools is provided for each of these methods in Table 1. Algorithms that exploit read-pair (1000 Genomes Project Consortium 2010; Mills et al. 2011; Quinlan et al. 2010; Keane, RetroSeq; Chen et al. 2009; Hormozdiari et al. 2009; Hormozdiari et al. 2010; Hormozdiari et al. 2011; Korbel et al. 2009; Lee et al. 2009; Qi and Zhao 2011; Zeitouni et al. 2010) can detect four types of SVs (deletions, insertions, inversions, and tandem duplications). They look for read-pairs that are anomalously aligned to the reference genome, e.g., reads that are either too far apart or in the wrong orientation. When one of the paired-reads is mapped to the reference genome and the other is unmapped, this suggests a large insertion.

**Table 1:** A summary of software tools to detect simple and complex SVs

Method	Software	Detectable SV types					Reference
		Del	Ins	Inv	Dup	Complex	
Read-pair	BreakDancer	✓	✓	✓	✓		(Chen et al. 2009)
	HYDRA	✓	✓	✓	✓		(Quinlan et al. 2010)
	inGAP-sv	✓	✓	✓	✓		(Qi and Zhao 2011)
	MoDIL	✓	✓	✓	✓		(Lee et al. 2009)
	PEMer	✓	✓	✓	✓		(Korbel et al. 2009)
	RetroSeq		✓				(Keane, RetroSeq)
	SPANNER	✓	✓	✓	✓		(1000 Genomes Project Consortium 2010; Mills et al. 2011)
	SVDetect	✓	✓	✓	✓		(Zeitouni et al. 2010)
	VariationHunter	✓	✓	✓	✓		(Hormozdiari et al. 2009; Hormozdiari et al. 2010; Hormozdiari et al. 2011)
Split-read	Dindel	✓	✓				(Albers et al. 2011)
	Pindel	✓	✓				(Ye et al. 2009)
	SplazerS	✓	✓				(Emde et al. 2012)
	Splitread	✓	✓				(Karakoc et al. 2011)
	SRiC	✓	✓				(Zhang et al. 2011)
Read depth	cnD	✓			✓		(Simpson et al. 2010)
	cn.MOPS	✓			✓		(Klambauer et al. 2012)
	CNVer	✓			✓		(Medvedev et al. 2010)
	CNVnator	✓			✓		(Abyzov et al. 2011)
	EWT	✓			✓		(Yoon et al. 2009)
Assembly	ABySS	✓	✓	✓	✓		(Simpson et al. 2009)
	ALLPATHS-LG	✓	✓	✓	✓		(Gnerre et al. 2011)
	EULER-USR	✓	✓	✓	✓		(Chaisson et al. 2009)
	NovelSeq	✓	✓	✓	✓		(Hajirasouliha et al. 2010)
	SOAPdenovo	✓	✓	✓	✓		(Li et al. 2010)
	TIGRA	✓	✓	✓	✓		(Mills et al. 2011)
Meta caller	SVMerge	✓	✓	✓	✓	✓	(Wong et al. 2010)

In the split-read approach (Albers et al. 2011; Emde et al. 2012; Karakoc et al. 2011; Ye et al. 2009; Zhang et al. 2011), one of the paired-reads is mapped to the reference genome, acting as an anchor, while the other encompasses the structural variant, typically a small insertion. Additionally, the high coverage of next-generation sequencing makes it possible to detect copy number changes using the read-depth approach (Abyzov et al. 2011; Klambauer et al. 2012; Medvedev et al. 2010; Simpson et al. 2010; Yoon et al. 2009). Assembly algorithms (Mills et al. 2011; Chaisson et al. 2009; Gnerre et al. 2011; Hajirasouliha et al. 2010; Li et al. 2010; Simpson et al. 2009) have the most power to detect SVs at base-pair resolution; however, they also miss considerable variation, especially at complex genomic regions (Alkan et al. 2011).

None of the algorithms is ideal, and none deals well with complex SVs that consist of a combination of rearrangements (such as an insertion abutting a deletion or an inversion within a gain) (reviewed in (Quinlan and Hall 2012)). In a recent study (Yalcin et al. 2012), we manually examined the whole of chromosome 19 for structural variation using the short read visualization tool LookSeq (Manske and Kwiatkowski 2009). We found greater diversity and complexity in SVs than had previously been reported. The manually curated set of SVs provided a benchmark for developing a method to call complex SVs at a genome-wide level (SVMerge (Wong et al. 2010)). It should be noted that SVMerge is the first tool, to date, that can effectively call complex SVs (Table 1). To study the full spectrum of SVs, future algorithms need to consider the complex forms of PEM patterns, described in (Yalcin et al. 2012).

## Functional impact of structural variation

Although twice more base pairs are affected as a consequence of structural variation than single nucleotide mutation, it remains unclear to what extent structural variants contribute to quantitative phenotypic differences. On the one hand, there have been some reports that common SVs are less likely than common SNPs to contribute to phenotypic variation (Keane et al. 2011; Conrad et al. 2010). On the other hand, several studies have provided remarkable estimates of the contribution of SVs to variation in transcript abundance: estimates ranged from 10 to 74 % (Yalcin et al. 2011; Cahan et al. 2009; Henrichsen et al. 2009; Stranger et al. 2007). It has also been reported that structural variants can influence gene expression up to 500 kbp from their margins (Henrichsen et al. 2009). Since gene expression variation is believed to contribute to variation in phenotypes at a whole-organism level (Schadt et al. 2005), results from these studies might indicate that the phenotypic impact of SVs is large.

Our genome-wide catalog of SVs was used in two ways to address the extent to which SVs affect phenotypic differences. The first used results from genome-wide association studies in an outbred population of mice, the Northport heterogeneous stock (HS) mice (Demarest et al. 2001; Talbot et al. 1999). The Northport HS mice are animals derived from eight of the sequenced strains (A/J, AKR/J, BALB/cJ, C3H/HeJ, C57BL/6J, CBA/J, DBA/2J, and LP/J) (Keane et al. 2011). Because many recombinants have accumulated since the creation of the HS population, mapping resolution of quantitative trait loci (QTLs) is high (to an average region of 3 Mbp). The HS population is not only unique for its high mapping resolution but also for the large number of QTLs that have already been mapped for a diversity of traits (about 100 traits) (Valdar et al. 2006). Since the HS mice are derived from eight fully sequenced strains, they can be used to assess the impact of genomic variants such as SVs on phenotypic differences (Yalcin and Flint 2012).

To do this, we applied a test of functionality (Yalcin et al. 2005) that allowed us to discriminate between SVs that are likely to be functional and those that are not. We found that the larger the effect, the more likely it is to arise from a structural variant (Keane et al. 2011). However, there are very few QTLs that are likely to be due to a structural variant: we identified just 12 QTLs where a structural variant overlapped a gene and where the effect size was in the top 5 % of the distribution. Table 2A lists these genes and the putative phenotypes with which they are associated. In one case, we used complementation (Mackay 2004) of a deletion of the *H2–Ea* promoter to confirm the effect of this SV on a T cell phenotype (Yalcin et al. 2010). In another case, we had evidence in favor of a causative role for an insertion in the promoter of one gene (*Eps15*) that abolished gene expression. We found that *Eps15* knockout mice exhibited a significantly lower locomotor activity compared to matched wild-type mice, indicating that the insertion is likely the cause of the QTL.

**Table 2:** Mouse genes affected by a structural variant correlated to a phenotype

Gene	SV event	Chr	SV start	SV stop	Phenotype	Reference
A. Genes with SV associated with quantitative traits						
*4921524J17Rik*	LINE Ins	8	87957244	87957245	Red cells: mean cellular volume	(Yalcin et al. 2011)
*Eps15*	IAP Ins	4	108951263	108951264	Home cage activity	(Yalcin et al. 2011)
*Fcer1a*	Ins	1	175158884	175158885	Mean platelet volume	(Yalcin et al. 2011)
*Gm6320*	Del	13	113783196	113783359	Hippocampus cellular proliferation	(Yalcin et al. 2011)
*Grin3a*	Del	4	49690362	49690363	Hippocampus cellular proliferation	(Yalcin et al. 2011)
*H2–Ea*	Del	17	34483681	34483682	T-cells: CD4/CD8 ratio	(Yalcin et al. 2011; Yalcin et al. 2010)
*Nnt*	Del	13	120164268	120164269	Glucose intolerance	(Freeman et al. 2006)
*Sec23b*	SINE Ins	2	144402760	144402971	OFT total activity	(Yalcin et al. 2011)
*Snrnp40*	SINE Ins	4	130038388	130038389	T-cells: % CD3	(Yalcin et al. 2011)
*Tmc3*	IAP Ins	7	90731819	90731820	Wound healing	(Yalcin et al. 2011)
*Tmem104*	Del	11	115106127	115106250	Serum urea concentration	(Yalcin et al. 2011)
*Trim30b*	Del	7	111504989	111505193	Red cells: mean cellular hemoglobin	(Yalcin et al. 2011)
*Trim5*	Ins	7	111397607	111479433	Red cells: mean cellular hemoglobin	(Yalcin et al. 2011)
B. Genes with SV affecting coding regions						
*Amd2*	Ins	18	64607747	64609669	Biosynthesis of polyamines	(Yalcin et al. 2011; Persson et al. 1999)
*Defb8*	Ins + Del	8	19447465	19450575	Infection and immunity	(Yalcin et al. 2011; Bauer et al. 2001)
*Fam110c*	VNTR	12	31759321	31759461	Cell migration	(Yalcin et al. 2011)
*Fcrl5*	Del	3	87245084	87245947	Infection and immunity	(Yalcin et al. 2011)
*Fv1*	Del	4	147244398	147245739	Infection and immunity	(Yalcin et al. 2011; Best et al. 1996)
*Klrb1a*	Del	6	128559593	128559740	Infection and immunity	(Yalcin et al. 2011)
*Klri2*	Del	6	129689526	129691211	Infection and immunity	(Yalcin et al. 2011)
*Krtap16*-*1*	VNTR	16	88874294	88874392	Hair formation	(Yalcin et al. 2011)
*Krtap5*-*5*	VNTR	7	149415121	149415210	Hair formation	(Yalcin et al. 2011)
*Nes*	VNTR	3	87780530	87780662	Brain development	(Yalcin et al. 2011)
*Nlrp1c*	Ins	11	71046193	71101410	Embryonic development	(Yalcin et al. 2011)
*Olfr1055*	IAP Ins	2	86179898	86186982	Olfactory	(Yalcin et al. 2011)
*Olfr234*	Del	15	98328544	98328861	Olfactory	(Yalcin et al. 2011)
*Olfr913*	Del	9	38402589	38403498	Olfactory	(Yalcin et al. 2011)
*Pglyrp3*	Del	3	91831862	91835385	Infection and immunity	(Yalcin et al. 2011)
*Rtp3*	VNTR	9	110889280	110889465	Bone density	(Yalcin et al. 2011)
*Skint4,3,9*	Ins	4	111731004	112272814	Infection and immunity	(Yalcin et al. 2011; Boyden et al. 2008)
*Soat1*	Del	1	158394620	158401436	Hair interior defects	(Yalcin et al. 2011; Wu et al. 2010)
*Tas2r103*	Del	6	132985563	132986696	Taste	(Yalcin et al. 2011; Nelson et al. 2005)
*Tas2r120*	Del + Ins	6	132580541	132613777	Taste	(Yalcin et al. 2011; Nelson et al. 2005)
*Trim5,12a*	Ins	7	111397607	111479433	Infection and immunity	(Yalcin et al. 2011; Tareen et al. 2009)
*Ugt2b38*	Del	5	87850554	87854999	Metabolism	(Yalcin et al. 2011)
*Zfp607*	Del	7	28646761	28671650	DNA-binding	(Yalcin et al. 2011)
*Zfp872*	VNTR	9	22004856	22005023	DNA-binding	(Yalcin et al. 2011)

The second way in which a genome-wide catalog of SVs can be used to assess the functional impact of SVs is by identifying variants that remove a coding segment of a gene, effectively creating a null or altered allele. Again these are relatively few. A summary of genes containing SVs affecting coding regions is provided in Table 2B. Most of these SVs have been newly identified (Yalcin et al. 2011), and a small number were already known (Best et al. 1996; Boyden et al. 2008; Nelson et al. 2005; Persson et al. 1999; Wu et al. 2010). These SVs, with large effects on a phenotype, are the equivalent of rare variants found in human populations. In the mouse, these SVs are rare relative to their abundance in the genome; however, they provide, for the first time, biological insights into the influence of these events on phenotype.

## Complex molecular architecture of SVs

Because of their high breakpoint accuracy, our genome-wide catalog of SVs not only expands knowledge of the molecular architecture of SVs, it also allows inferring a SV mechanism of formation with a high degree of precision. We know that more than half of the SVs are caused by retrotransposition of LINEs (25 % of SVs), SINEs (short interspersed nuclear elements; 15 %), and LTRs (long terminal repeats; 14 %), followed by VNTRs (variable-number tandem repeats; 15 %), and pseudogenes (2 %) (Yalcin et al. 2011).

By characterizing the sequence features around SV breakpoints, we found that about a quarter of SVs have smaller rearrangements at their breakpoints, such as a microinsertion or a microdeletion at the breakpoint of a larger variant (Yalcin et al. 2012). For example, two alleles have been reported for a β-defensin gene (*Defb8*) that differ by 3 bp changes in the second exon (Bauer et al. 2001; Taylor et al. 2009). We found that, in fact, these documented exonic changes are linked to a previously undetected 3,192 bp deletion (Yalcin et al. 2011).

It is acceptable to assume that the complex molecular architecture of SV (microstructures at SV breakpoints) will correlate with complex mechanisms of SV formation. Two mechanisms, a DNA replication fork stalling and template switching (FoSTeS) and a microhomology-mediated break-induced replication (MMBIR), have been proposed to generate such complex SVs in the human genome (Zhang et al. 2009). It could be that the complex SVs we see in the mouse genome (about 25 % of SVs) have also formed through mutational forces during DNA replication.

However, as highlighted previously, there are real limitations in the methods of SV detection with complex molecular architecture. Ideally, sequencing of larger fragments of DNA or, even better, complete de novo assembly of the genome would typically be required to resolve the full spectrum of complex architecture of structural variants.

## Prospects for full-genome sequences

While our high-resolution catalogs of sequence variation advanced studies correlating genotype to phenotype, the ultimate goal is to obtain the complete genomic sequence of all common laboratory mouse strains. As a first step toward this goal, the sequencing reads generated by the Mouse Genomes Project have been put through de novo assembly using the Velvet assembler (Zerbino and Birney 2008) and preliminary draft genomes are available for download from the project FTP site (see Box 1). Preliminary analysis of these draft assemblies shows that approximately 90 % of the coding regions of the strains can be found in the assemblies of the strains, although this number is lower for the wild-derived strains. Clearly much work remains to bring these assemblies up to a standard approaching that of the C57BL/6J reference genome.

**Box 1:** 

Resource name	Description	URL
Mouse genomes project	Wellcome Trust Sanger Institute mouse genomes project webpage with details of the mouse strains sequenced and how to get the data	http://www.sanger.ac.uk/resources/mouse/genomes/
Mouse genomes project browser	The website for querying and downloading lists of SNPs, indels, and structural variants	http://www.sanger.ac.uk/cgi-bin/modelorgs/mousegenomes/snps.pl
dbSNP	All SNP and indel variants from the 17 strains have been submitted to dbSNP under handle “SC_MOUSE_GENOMES”	http://www.ncbi.nlm.nih.gov/projects/SNP/
DGVa	Database of genomic variants archive (DGVa) is a repository that provides archiving, accessioning, and distribution of publicly available genomic structural variants. All structural variants from the 17 strains have been submitted under accession estd118 and estd185	http://www.ebi.ac.uk/dgva/

## Future work

The Mouse Genomes Project produced an unprecedented amount of raw sequencing data across 17 mouse strains. The analysis of these data has painted the most comprehensive picture of molecular variation in the mouse genome to date. However, due to limitations in second-generation sequencing technologies, up to 30 % more sequence variation remains to be discovered. To this end, efforts are underway to resequence the strains with longer and higher-quality reads from newer versions of second-generation sequencing technologies. However, the key to discovering the complete set of sequence variants will be the development of third-generation sequencing technologies capable of producing much longer read sequences (multiple kbp in size) so that we can interrogate parts of the genome that are out of reach for current technologies (Schadt et al. 2010).

The ultimate goal of the de novo assembly efforts is to produce full-genome sequences of the 17 strains of quality comparable to that of the reference genome. It has been shown that to go from a set of assembled contigs to larger scaffolds of hundreds of kilobases, sequencing from the ends of large fragments of varying sizes is required (Gnerre et al. 2011). Long-fragment sequencing remains challenging with second-generation sequencing technologies; primarily due to difficulties in producing sufficiently diverse sequencing libraries and reliable methods are still under development (Van Nieuwerburgh et al. 2012). De novo assembly is an area that will benefit greatly from the development of third-generation sequencing technologies capable of producing much longer read lengths.

The C57BL/6J mouse has been the mouse reference genome since the turn of century. However, as we produce improved de novo assemblies from the newly sequenced strains, we can use the novel sequence haplotypes found in subsets of the 17 strains and not found in the reference genome to define the mouse pan-genome reference (Dunn et al. 2012; Li et al. 2010; Muzzi and Donati 2011). The goal of creating this pan-genome would be to reduce the reference bias that affects many experiments and allow for the discovery of sequence variation shared among subsets of strains and not found in C57BL/6J.
